# Maceration determines diagnostic yield of fetal and neonatal whole body post‐mortem ultrasound

**DOI:** 10.1002/pd.5615

**Published:** 2019-11-29

**Authors:** Susan Cheng Shelmerdine, Dean Langan, Uday Mandalia, Neil James Sebire, Owen John Arthurs

**Affiliations:** ^1^ Department of Clinical Radiology Great Ormond Street Hospital for Children London UK; ^2^ UCL Great Ormond Street Institute of Child Health Great Ormond Street Hospital for Children London UK; ^3^ Department of Histopathology Great Ormond Street Hospital for Children London UK

## Abstract

**Objectives:**

To determine factors in nondiagnostic fetal and neonatal post‐mortem ultrasound (PMUS) examinations.

**Methods:**

All fetal and neonatal PMUS examinations were included over a 5‐year study period (2014‐2019). Nondiagnostic image quality by body parts (brain, spine, thorax, cardiac, and abdomen) was recorded and correlated with patient variables. Descriptive statistics and logistic regression analyses were performed to identify significant factors for nondiagnostic studies.

**Results:**

Two hundred sixty‐five PMUS examinations were included, with median gestational age of 22 weeks (12‐42 wk), post‐mortem weight of 363 g (16‐4033 g), and post‐mortem interval of 8 days (0‐39 d).

Diagnostic imaging quality was achieved for 178/265 (67.2%) studies. It was high for abdominal (263/265, 99.2%), thoracic (264/265, 99.6%), and spine (265/265, 100%) but lower for brain (210/265, 79.2%) and cardiac imaging (213/265, 80.4%). Maceration was the best overall predictor for nondiagnostic imaging quality (*P* < .0001). Post‐mortem fetal weight was positively associated with cardiac (*P* = .0133) and negatively associated with brain imaging quality (*P* = .0002). Post‐mortem interval was not a significant predictor.

**Conclusions:**

Fetal maceration was the best predictor for nondiagnostic PMUS, particularly for brain and heart. Fetuses with marked maceration and suspected cardiac or brain anomalies should be prioritised for post‐mortem MRI.

**Figure 1:**
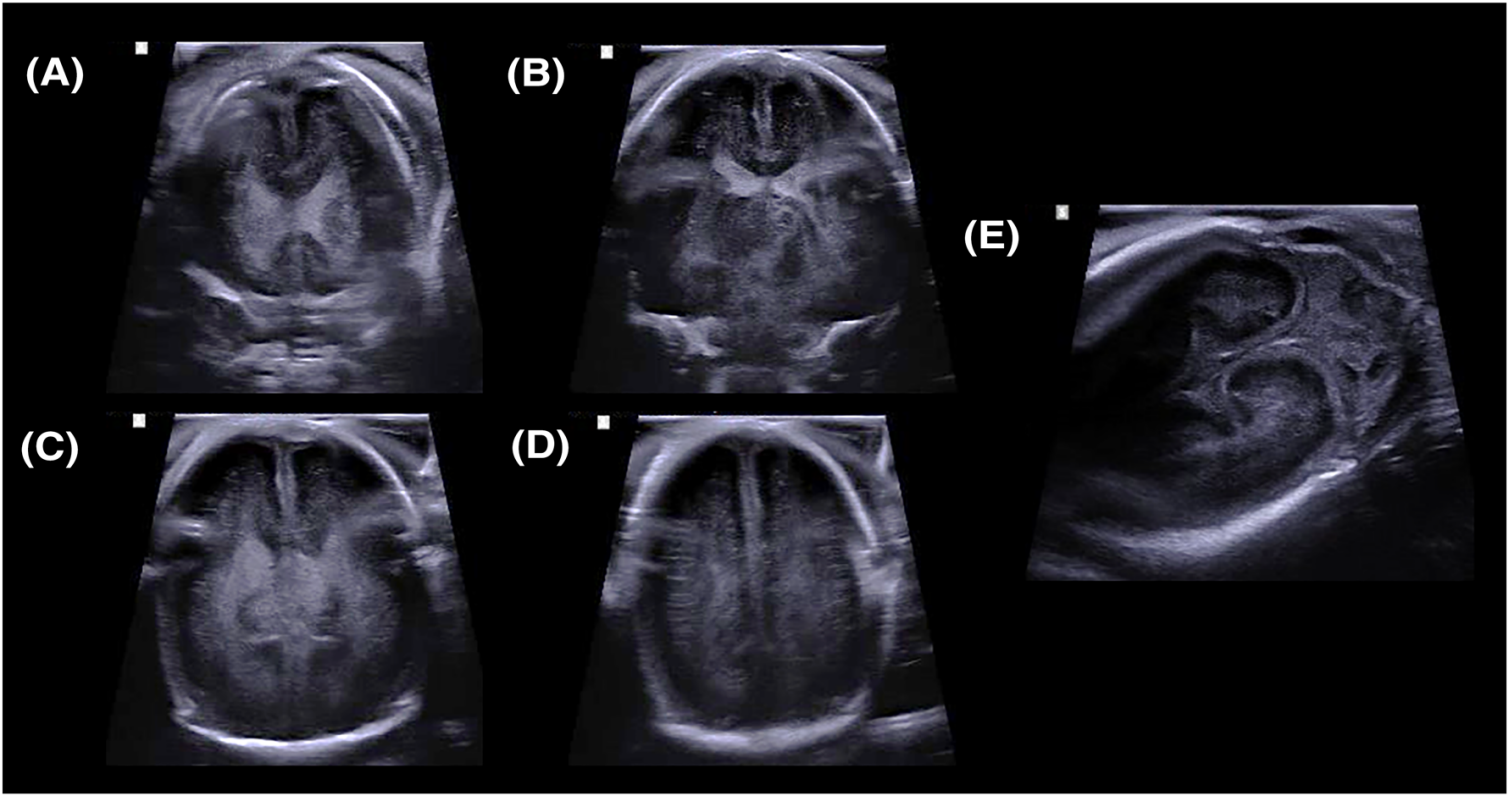
Example of a diagnostic quality post‐mortem ultrasound image of the brain in a 17‐week gestational aged fetus, obtained in a water bath. Coronal ultrasound images through A, the frontal horns of the lateral ventricles, B, the body of the lateral ventricles, C, the trigone of the lateral ventricles, and D, the occipital lobes respectively. Image (E) demonstrates image acquisition via the mastoid fontanelle, depicting the cerebellar hemispheres in coronal section [Colour figure can be viewed at http://wileyonlinelibrary.com]

**What is already known about this topic?**

- Post‐mortem ultrasound is a feasible technique for fetal and neonatal death investigation.
- When imaging quality is diagnostic, there is a similar diagnostic accuracy rate between fetal and neonatal post‐mortem ultrasound and MRI.

**What does this study add?**

- The brain and heart are the most likely body parts to be nondiagnostic at post‐mortem ultrasound examination.
- Maceration is the most significant contributing factor for nondiagnostic quality ultrasound imaging
- Fetal and neonatal deaths should be prioritised for post‐mortem MRI where they are macerated and/or where there is a high antenatal suspicion for cardiac or brain abnormalities.

**Abbreviations**
LIA: less invasive autopsy
MIA: minimally invasive autopsy
MinIMAL: minimally invasive autopsy with laparoscopic‐assisted sampling
PMI: post‐mortem interval (ie, time between death and imaging)
PMMR: post‐mortem magnetic resonance imaging
PMUS: post‐mortem ultrasound
VACTERL: vertebral, anorectal, cardiac, tracheo‐oeosphageal fistulae, renal, limb anomalies
VSD: ventricular septal defect

## 1 INTRODUCTION

Low parental consent rates and acceptability for conventional autopsy techniques^1^, ^2^ have resulted in the development of non‐invasive alternatives.^3^, ^4^, ^5^, ^6^ This has centred predominantly on radiological imaging, with perinatal post‐mortem MRI (PMMR) demonstrating high concordance rates (90%) when compared with autopsy as reference standard,^7^ with similarly successful results replicated in different centres internationally.^8^, ^9^ Whilst this is welcome news for many parents, access to PMMR may be limited, and increasing clinical pressures have reduced the availability of scanner time in many centres.

Recent literature has suggested that post‐mortem ultrasound (PMUS) in the perinatal population could offer a potential alternative imaging solution.^10^ One recent study even suggested that fetal and neonatal PMUS could provide the same diagnostic accuracy for cause of death as 3T PMMR, when the images were of diagnostic quality.^11^ With ultrasound imaging being more affordable and less time‐consuming (approximately 20 min for PMUS^12^ versus 45‐90 min for PMMR^13^), one potential imaging protocol may be to use PMUS as a triage tool for non‐invasive autopsies (NIAs), whilst referring only complex cases or nondiagnostic PMUS studies for PMMR, thus saving time and clinical resources. This pathway could be streamlined further if it was possible to predict certain variables or patient factors that would render a nondiagnostic quality PMUS and “fast‐track” these cases for PMMR.

In this study, our objective was therefore to identify best demographic or patient clinical predictors for achieving a diagnostic quality PMUS examination, in a large fetal and neonatal post‐mortem cohort.

## 2 METHODS

Ethical approval was granted for this prospective, single centre cohort study (REC 09/H0713/2) and all samples handled in accordance with the Human Tissue Act (2004). Parental written consent for post‐mortem imaging was obtained in each case.

### 2.1 Patient selection

Consecutive unselected fetal and neonatal deaths, with parental consent for post‐mortem imaging were included over a 5‐year period spanning 1 July 2014 to 1 July 2019. All cases were deemed eligible for inclusion. We excluded from analysis cases in whom whole body ultrasound had not been performed.

### 2.2 PMUS imaging

All PMUS was performed by one of two board‐certified paediatric radiologists, with 2 years (UM) and 4 years (SCS) of specialist post‐mortem radiology and paediatric ultrasound scanning experience. The radiologists were only given the gestational age of the patient and suspected mode of delivery or death. They were blinded to all other clinical history details (including results of antenatal imaging tests). PMUS was conducted according to published guidelines,^12^, ^14^ and each study took approximately 20 minutes to complete. All bodies were stored and refrigerated at 4°C in the hospital mortuary prior to imaging.

Ultrasound was performed using a high‐frequency linear probe (7‐16 MHz) for all body parts, on a dedicated ultrasound machine (Samsung, model HM70A), reported using a predefined template, adapted from the ISUOG fetal imaging guidelines^15^, ^16^, ^17^ (see Appendix A) for comment across five different body parts (brain, spine, chest, cardiac, and abdomen) and for overall diagnosis. Suboptimal image quality or inability to perform adequate views were defined as “nondiagnostic” and judged by the radiologist performing the study according to the normal expected appearances for the fetal gestational age.^18^, ^19^, ^20^

**Figure 2:**
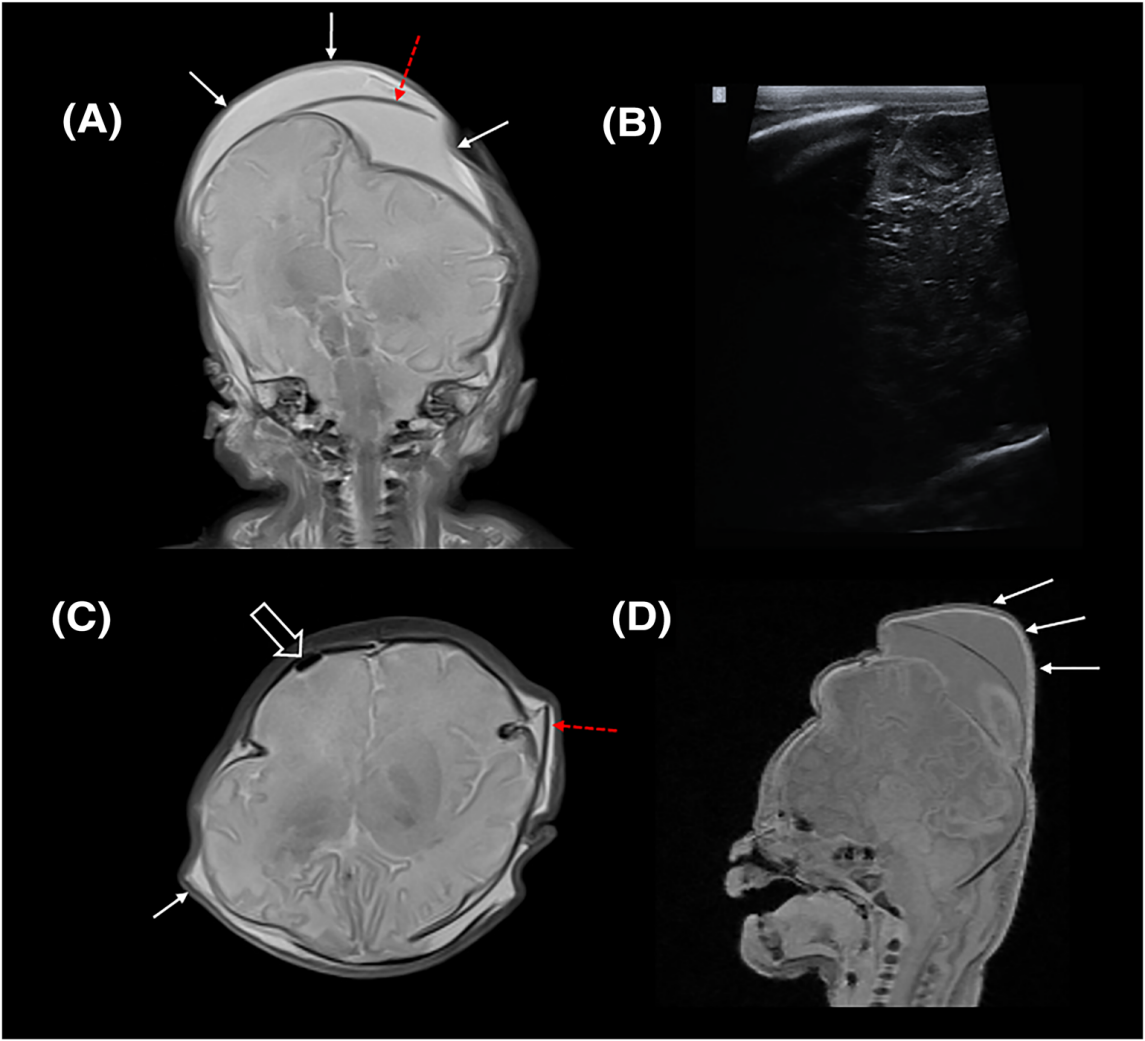
Nondiagnostic post‐mortem ultrasound brain imaging in a 38‐week gestational aged fetus. A, Coronal T2‐weighted post‐mortem MRI image is compared with B, coronal ultrasound image, demonstrating poor sonographic penetrance and visualisation of the right cerebral hemisphere. The MRI image demonstrates marked soft tissue scalp oedema (white arrows) and cranial sutures overlapping (red dashed arrow) contributing to the poor ultrasound quality. Further, C, axial T2‐weighted and D, sagittal T1‐weighted MRI images reveal a locule of intracranial gas (open arrow) and further areas of oedema (white arrow) and sutural disruption (red dashed arrow) [Colour figure can be viewed at http://wileyonlinelibrary.com]

**Figure 3:**
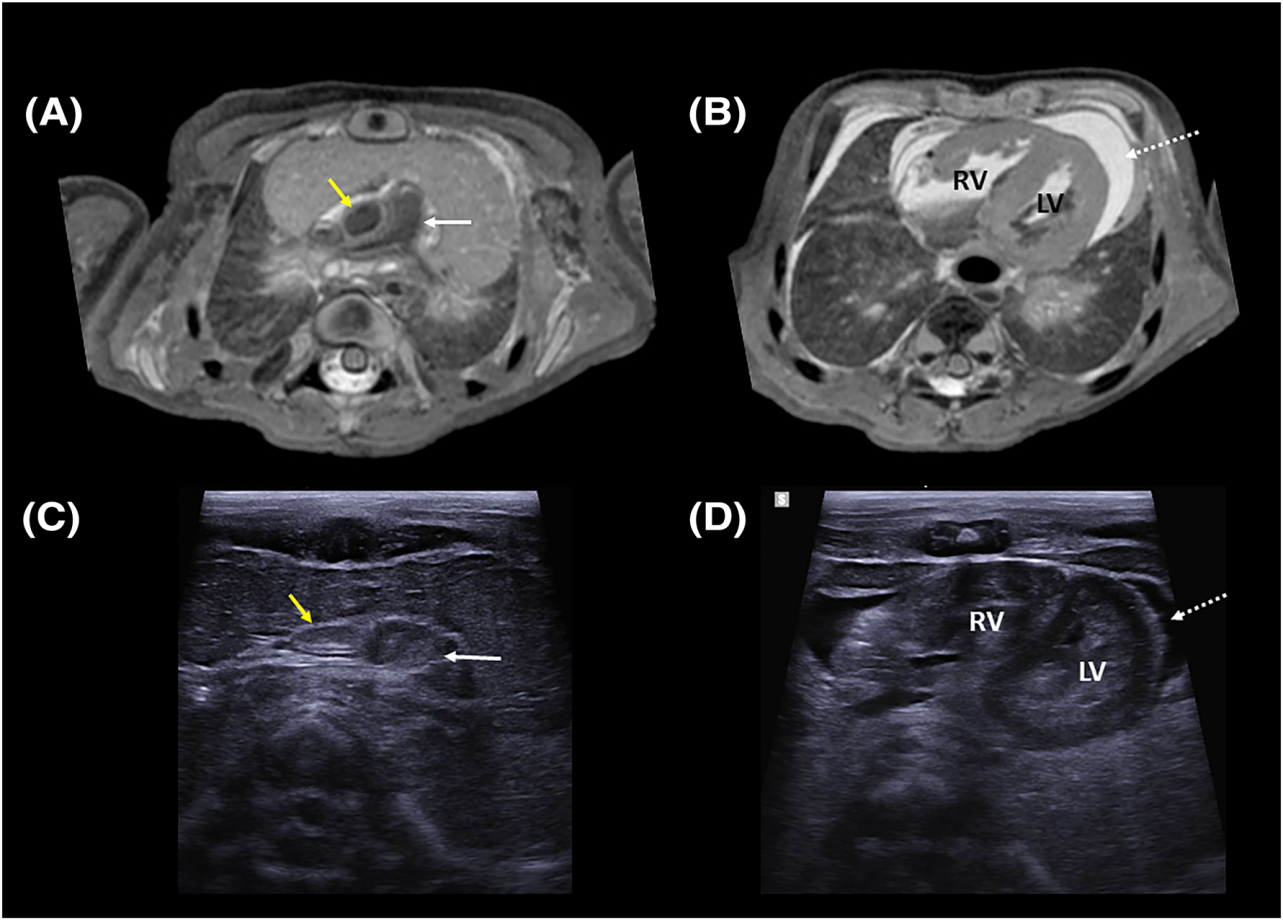
An example of diagnostic quality post‐mortem ultrasound images in a 37‐week gestational aged fetus. The axial T2 weighted post‐mortem MRI images through A, the cardiac outflow tracts and B, the long axis of the heart are compared with transverse ultrasound images in C, and D, These show how several anatomical structures including the main pulmonary artery (white arrow), ascending aorta (yellow arrow), right ventricle (RV), and left ventricle (LV) are visualised in both modalities. A large pericardial effusion (dashed arrow) is also visualised on both MRI and ultrasound [Colour figure can be viewed at http://wileyonlinelibrary.com]

**Figure 4:**
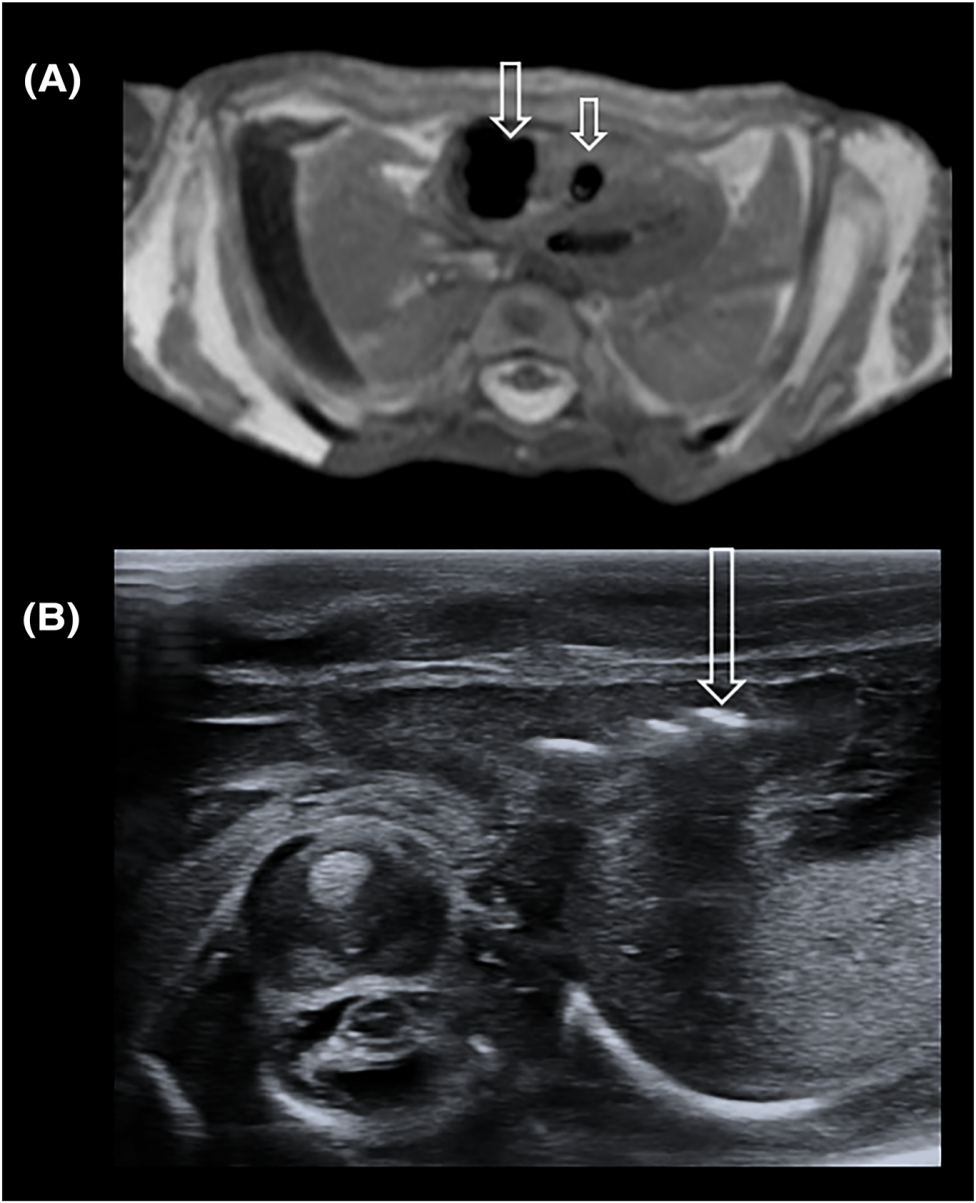
Nondiagnostic post‐mortem ultrasound cardiac images in a 25‐week gestational aged fetus. (A) Axial T2‐weighted post‐mortem MRI of the heart reveals multiple gas locules in the right atrium and ventricle (open arrows), which obscure sonographic wave penetrance and thus visualisation of the cardiac chambers on the (B) transverse ultrasound image. On the ultrasound image, intracardiac locules of gas are identified as white linear echogenic artefacts with posterior acoustic shadowing (open arrow) [Colour figure can be viewed at http://wileyonlinelibrary.com]

As an example, a diagnostic brain PMUS study should allow for visualisation of both cerebral hemispheres, ventricles and the posterior fossa through the anterior, sphenoid, and mastoid fontanelles (Figure 1); however, a nondiagnostic brain PMUS would preclude these key anatomical landmarks (Figure 2). Diagnostic thoracic studies should allow for the unaerated lungs and diaphragm to be outlined. Diagnostic cardiac studies should allow for the four cardiac chambers and outflow tracts to be delineated (as in Figure 3); however, the presence of intracardiac gas (Figure 4), and any distortion or compressibility of the chest could be deemed nondiagnostic. Abdominal PMUS imaging should allow for the liver, spleen, kidneys, bladder, bowel, and genitalia to be visualised and imaging of the spine should allow for identification of the entire spinal cord, including the conus medullaris and the bony sacrum to rule out neural tube or sacral defects.

### 2.3 Data collation and collection

All results were transferred prospectively into an imaging database in Excel (version 14.7.3, Microsoft, Seattle, USA). No additional re‐review of the original PMUS images was performed.

From the finalised autopsy report, conducted by one of seven specialist consultant paediatric pathologists at our centre, the demographic details and biometric measurements taken at the external examination of the autopsy were also entered into the database, including the following:

- Patient gender (male, female, or undetermined);
- Gestational age (weeks);
- Post‐mortem weight (g);
- Crown‐rump length (CRL) (cm);
- Crown‐heel length (CHL) (cm);
- Head circumference (HC) (cm);
- Post‐mortem interval (PMI, days) (ie, the time between death [for neonatal deaths] or delivery [for terminations of pregnancy, stillbirths and intrauterine fetal deaths] and the PMUS);
- Mode of death/delivery; and
- Maceration score (maceration is the process of tissue autolysis, which occurs during intrauterine fetal retention.^21^ Given that the exact timing of fetal demise in utero is difficult and therefore, the length of intrauterine retention, a standard pathologist assessment of the body at external examination was scored from 0 = no maceration to 3 = severe/established maceration, as per previous publications^22^, ^23^).

### 2.4 Data analysis

We used “diagnostic image quality” (yes/no) as the main outcome measure according to the five body parts assessed (brain, spine, cardiac, thoracic, and abdominal) and also for all organ systems combined (whole body assessment—scored as “diagnostic” if all five body parts were deemed to be of “diagnostic ultrasound image quality”).

Total numbers and percentages are presented of diagnostic ultrasound studies for each body systems and descriptive statistics for other demographic variables. Where there were sufficient numbers of both diagnostic and nondiagnostic cases for review, logistic regression (both univariable and multivariable analysis) was used to investigate how the odds of a diagnostic quality study changes according to demographic variables (eg, gender, post‐mortem weight, gestational age, PMI, CRL, CHL, HC, and maceration score). The estimated odds were converted to “probabilities of diagnostic quality” for graphical display, with 95% confidence intervals for these predicted probabilities. Goodness‐of‐fit statistics (the Akaike [AIC] and Bayesian information criterion [BIC]) were used, as well as clinical reasoning, to decide on which demographic variables were included in further multivariable models. All analyses were performed with R v2.15.2, and all estimates were presented with 95% confidence intervals derived from the profile likelihood method.

## 3 RESULTS

### 3.1 Study cohort

In total, 275 fetuses underwent a PMUS examination during the 5‐year study period. Of these, 10/275 (3.6%) were excluded due to an incomplete examination. The final study cohort therefore composed of 265 complete whole body PMUS examinations.

Case demographics are outlined in Tables 1 and 2. The median gestational age was 22 weeks (range 12‐42), with median PMI time of 8 days (range 0‐39; time between delivery and death to ultrasound examination). In 262/265 (98.9%) cases, the fetus was either delivered without signs of life or died on the same day as their birth (ie, age = 0 d). In three cases, the fetus survived for less than 28 days (aged 10, 19, and 28 d). The majority of our cases were the result of a termination of pregnancy (101/265, 38.1%) or miscarriage (88/265, 33.2%), and approximately one‐quarter of our fetuses demonstrated marked/extensive maceration (71/265, 26.8%).

**Table 1:** Case demographics for the continuous variables measured in our study cohort

	Median	Range	IQR
Gestational age, weeks	22	12‐42	19‐28
Post‐mortem interval, days	8	0‐39	5‐11
Post‐mortem weight, g	363	16‐4033	174‐1000
Head circumference, cm	18.0	2.2‐37.0	13.9‐25.0
Crown‐rump length, cm	18.8	6.0‐41.2	14.7‐25.9
Crown‐heel length, cm	27.1	8.2‐55.1	21.9‐35.6

Abbreviation: IQR, interquartile range.

**Table 2:** Demographic information for the categorical data variables measured in our study cohort, and proportion of those with a diagnostic quality whole body PMUS examination

	Total Number	Percentage, % (n = 265)	Whole Body PMUS Diagnostic Rate, %
Gender			
Male	152	57.4	73.0 (111/152)
Female	111	41.9	56.7 (66/111)
Indeterminate	2	0.8	50.0 (1/2)
Mode of death/demise			
Termination of pregnancy	101	38.1	78.2 (79/101)
Miscarriage	88	33.2	64.8 (57/88)
Intrauterine fetal death/stillbirth	66	24.9	51.5 (34/66)
Neonatal (0‐28 d)	10	3.8	80.0 (8/10)
Maceration score			
0, none	107	40.4	88.8 (95/107)
1, mild	55	20.8	72.7 (40/55)
2, moderate	32	12.1	62.5 (20/32)
3, marked/extensive	71	26.8	32.4 (23/71)

Abbreviations: CI, confidence interval; PMUS, post‐mortem ultrasound.

### 3.2 Diagnostic quality studies

For the whole body examination 178/265 (67.2%) of PMUS, studies were of diagnostic imaging quality for all five body parts. For a nonmacerated fetus (score = 0), 88.8% (95/107) examinations were of diagnostic quality overall, compared with 32.4% (23/71) of fetuses with marked/extensive maceration (score = 3). The proportion of whole body diagnostic quality examinations in different patient variables are outlined Table 2.

When looking at the individual body parts, all spinal ultrasound images (265/265, 100%), almost all thoracic (264/265, 99.6%), and abdominal ultrasound imaging studies (263/265, 99.2%) were of diagnostic quality. Ultrasound imaging of the brain (210/265, 79.2%) and heart (213/265, 80.4%) yielded the lowest diagnostic accuracy rates. The diagnostic ultrasound imaging rates with 95% confidence intervals for individual and whole body parts/systems are provided in Table 3.

**Table 3:** Diagnostic ultrasound imaging rates for each body part/system and all body parts/systems overall

	Total Number (n = 265)	Diagnostic Rate, % (95% CI)
Body part/system		
Brain	210	79.2 (73.8‐83.9)
Spine	265	100 (98.2‐100.0)
Thoracic	264	99.6 (97.6‐100.0)
Cardiac	213	80.4 (75.0‐84.9)
Abdominal	263	99.2 (97.0‐99.9)
All body parts/systems	178	67.2 (61.1‐72.7)

Abbreviation: CI, confidence interval.

### 3.3 Logistic regression analysis

Given that all of the spinal imaging was diagnostic and only a small number of abdominal (n = 2) and thoracic (n = 1) studies were nondiagnostic, further statistical analyses were conducted only for the assessment of the brain, cardiac, and all body parts overall.

In reviewing ultrasound imaging of the five body parts altogether, the multivariable model demonstrated that maceration was the most significant predictor for nondiagnostic image quality (Table 4), demonstrating a clear negative association with the odds of diagnosis. For example, a marked/extensive maceration score has an odds ratio of 0.061 (95% CI, 0.027‐0.128; *P* < .0001) compared with no maceration (score = 0). In other words, the odds of an “acceptable quality” ultrasound in a setting with no maceration are 16.5 times greater (1/0.061) than in a setting with severe maceration (95% CI, 7.81‐37.4). The probabilities of acceptable quality for each maceration score are given in Table 2 (eg, 88.5% for no maceration).

**Table 4:** Logistic regression analysis for diagnostic quality “whole body” imaging at post‐mortem ultrasound (PMUS) (ie, encompassing an overall diagnostic quality for all five body parts/systems recorded)

Case Variables	Univariable			Multivariable		
	OR	95% CI	*P* value	OR	95% CI	*P* value
Gender (ref = Female)	1.846	1.097‐3.119	0.0212	‐	‐	‐
Post‐mortem weight (per 100 g)	0.950	0.925‐0.976	0.0002	‐	‐	‐
Gestational age, wk	0.950	0.918‐0.983	0.0037	‐	‐	‐
Post‐mortem interval, d	0.973	0.923‐1.025	0.2965	‐	‐	‐
Crown‐rump length, cm	0.964	0.934‐0.995	0.0242	‐	‐	‐
Crown‐heel length, cm	0.973	0.952‐0.996	0.0217	‐	‐	‐
Head circumference, cm	0.980	0.947‐1.015	0.2524	‐	‐	‐
Maceration score (ref = 0):						
1, mild	0.337	0.142‐0.781	0.0115	0.337	0.142‐0.781	0.0115
2, moderate	0.210	0.082‐0.536	0.0011	0.210	0.082‐0.536	0.0011
3, marked/extensive	0.061	0.027‐0.128	<0.0001	0.061	0.027‐0.128	<0.0001
Mode of death (ref = TOP):						
IUD/stillbirth	0.296	0.149‐0.577	0.0004	‐	‐	‐
Miscarriage	0.512	0.266‐0.970	0.0416	‐	‐	‐
Neonatal	1.114	0.256‐7.734	0.8961	‐	‐	‐

Abbreviations: CI, confidence interval; OR, odds ratio.

For brain imaging, the multivariate model showed that post‐mortem weight (*P* = .0013), HC (*P* = .0288), and maceration score (eg, score 3 vs score 0, *P* < .0001) were all significant variables associated with image quality (Table 5).

**Table 5:** Logistic Regression analysis for diagnostic quality brain imaging at post‐mortem ultrasound

Case Variables	Univariable			Multivariable		
	OR	95% CI	*P* value	OR	95% CI	*P* value
Gender (ref = Female)	1.635	0.896‐2.996	0.1090	‐	‐	‐
Post‐mortem weight (per 100 g)	0.950	0.925‐0.976	0.0002	0.888	0.822‐0.950	0.0013
Gestational age, wk	0.921	0.886‐0.957	<0.0001	‐	‐	‐
Post‐mortem interval, d	0.958	0.904‐1.016	0.1440	‐	‐	‐
Crown‐rump length, cm	0.934	0.900‐0.968	0.0002	‐	‐	‐
Crown‐heel length, cm	0.951	0.926‐0.976	0.0002	‐	‐	‐
Head circumference, cm	0.956	0.918‐0.994	0.0238	1.114	1.017‐1.234	0.0288
Maceration score (ref = 0):						
1, mild	0.169	0.036‐0.615	0.0112	0.172	0.036‐0.642	0.0136
2, moderate	0.103	0.021‐0.398	0.0017	0.131	0.025‐0.547	0.0072
3, marked/extensive	0.026	0.006‐0.079	<0.0001	0.028	0.006‐1.234	<0.0001
Mode of death (ref = TOP):						
IUD/stillbirth	0.169	0.072‐0.373	<0.0001	‐	‐	‐
Miscarriage	0.427	0.179‐0.967	0.0456	‐	‐	‐
Neonatal	0.989	0.159‐19.19	0.9920	‐	‐	‐

Abbreviations: CI, confidence interval.

For cardiac imaging, the multivariable model showed that increased maceration scores (scores 2 and 3 vs score 0, *P* = .0104 and *P* < .0001, respectively) and increasing CRL (*P* < .0001) were significant predictors negatively associated with image quality (Table 6).

**Table 6:** Logistic Regression analysis for diagnostic quality cardiac imaging at post‐mortem ultrasound (PMUS)

Case Variables	Univariable			Multivariable		
	OR	95% CI	*P* value	OR	95% CI	*P* value
Gender (ref = Female)	2.090	1.129‐3.917	0.0197	‐	‐	‐
Post‐mortem weight (per 100 g)	1.056	1.016‐1.109	0.0133	‐	‐	‐
Gestational age, wk	1.056	1.009‐1.111	0.0268	‐	‐	‐
Post‐mortem interval, d	0.982	0.926‐1.047	0.5650	‐	‐	‐
Crown‐rump length, cm	1.074	1.028‐1.127	0.0021	‐	‐	‐
Crown‐heel length, cm	1.054	1.021‐1.091	0.0017	1.076	1.041‐1.117	<0.0001
Head circumference, cm	1.081	1.032‐1.137	0.0014	‐	‐	‐
Maceration score (ref = 0):						
1, mild	0.630	0.221‐1.858	0.3864	0.469	0.160‐1.420	0.1679
2, moderate	0.328	0.111‐0.997	0.0432	0.229	0.074‐0.725	0.0104
3, marked/extensive	0.133	0.055‐0.294	<0.0001	0.083	0.032‐0.197	<0.0001
Mode of death (ref = TOP):						
IUD/stillbirth	0.827	0.341‐2.059	0.6762	‐	‐	‐
Miscarriage	0.317	0.148‐0.649	0.0022	‐	‐	‐
Neonatal	1.330	0.221‐25.54	0.7948	‐	‐	‐

Abbreviations: CI, confidence interval.

**Figure 5:**
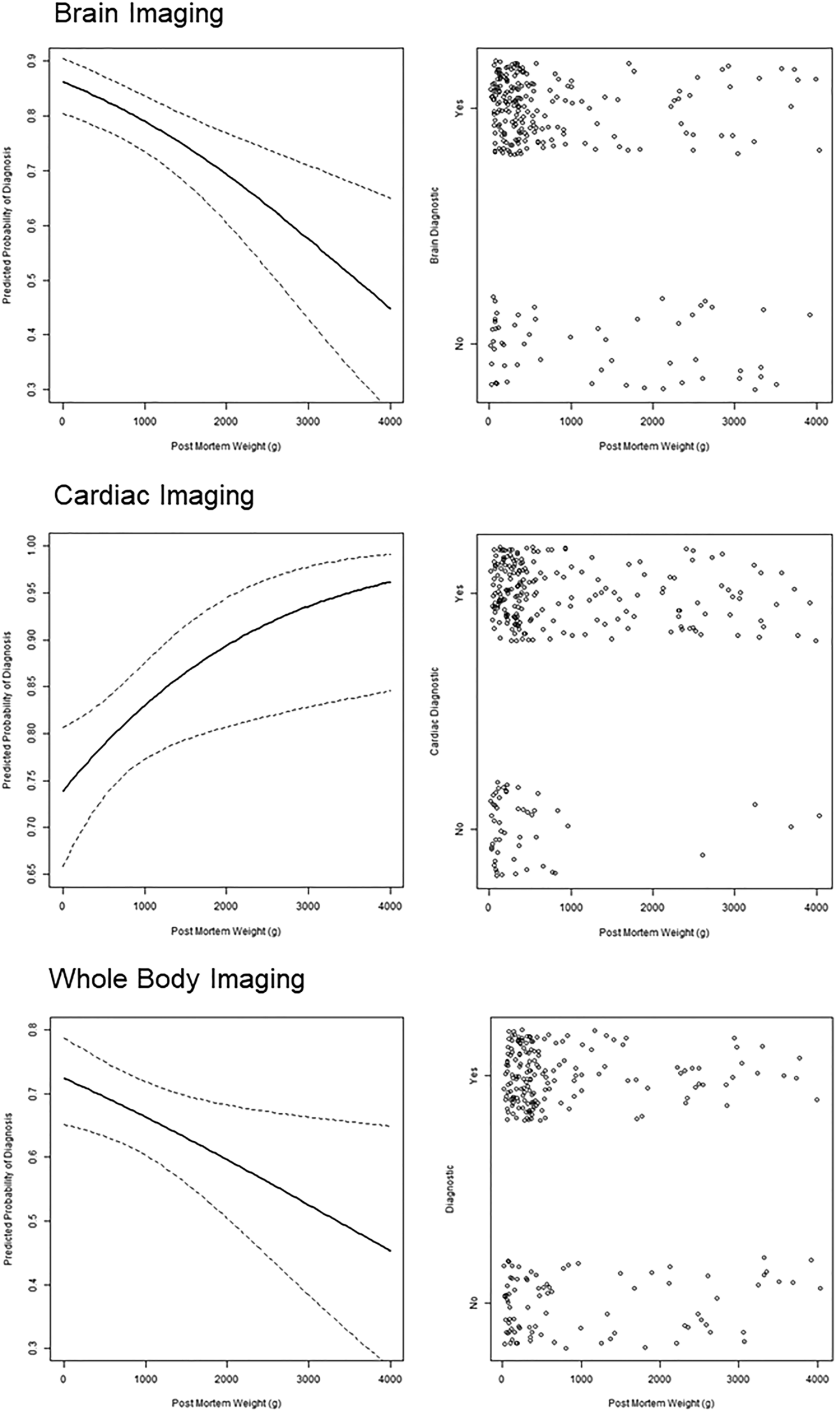
Graphical representation illustrating the relationship between post‐mortem weight (g) with predicted probability of diagnostic quality imaging (left) and presence of diagnostic quality imaging (right) for the brain, heart, and whole body overall. For the graphs on the left, the solid trend lines demonstrate the predicted probabilities, with the dotted lines representing 95% confidence intervals. On the right, each point on the graph represents a single examination in our study cohort (n = 265). With increasing post‐mortem weight, there is an increase in the probability of diagnostic image quality cardiac imaging but reduction in brain and whole body imaging quality

A graphical representation showing the changes in predicted diagnostic image quality and increase in post‐mortem body weight for brain, cardiac, and whole body ultrasound examinations are provided in Figure 5 (derived from the univariable models).

## 4 DISCUSSION

This study demonstrates that whilst the majority of fetal and neonatal PMUS studies are of diagnostic quality for all body parts, the most significant predictor for poor diagnostic quality was severe maceration. The most likely body parts to be nondiagnostic were the brain and the heart. In our multivariable analyses, we found that increasing CRL was positively associated for image quality of the heart but not the brain. We interpret this data to mean that macerated fetuses with an antenatally suspected cardiac or brain malformation should be prioritised for PMMR. Nonmacerated fetuses or those with a low likelihood of anomalies could still benefit from a PMUS in the first instance.

The maceration process begins immediately after intrauterine fetal death (IUFD) and is therefore impossible to prevent to preserve image quality and fetal integrity. This timing is not possible to determine, unless induced through termination of pregnancy. During the maceration process, enzymatic autolysis of cells and tissues leads to fetal epidermal desquamation, skin reddening, and oedema of the internal organs.^24^ The severity and extent of these changes are graded by pathologists at external assessment of the body and believed to correlate with the length of the intrauterine retention of the fetus.^24^, ^25^ This rapid degradation process, occurring at maternal body temperature, is in contrast to the presumed slower tissue breakdown after fetal delivery prior to autopsy (ie, the postmortem interval), where the body is typically preserved in a refrigerated environment. Fixation of the fetus in formalin post‐delivery (as performed by some authors^26^) can prevent the minimal further tissue breakdown in the PMI but does not reverse the maceration process that has already undertaken.

The results from this study, the largest cohort of fetal and neonatal PMUS examinations published to date, are consistent with previous studies that have consistently reported that brain and cardiac imaging are the most likely body parts to have a nondiagnostic result and that maceration plays a significant role. In these smaller studies, a nondiagnostic brain examination was seen in 18.6% (13/70),^14^ 18.7% (20/107),^27^ and 6.5% (4/62)^28^ of cases (compared with 20.7% [55/265] in this study); and a nondiagnostic cardiac examination was found in 19.7% (24/122),^14^ 33.6% (33/107),^27^ and 2.3% (2/86)^28^ (compared with 19.6%, 52/254 in this study). In one study,^29^ maceration was found to be associated with a nondiagnostic brain but not cardiac PMUS; however, gestational age and mode of delivery/death was not a factor for either. In another study by the same research group,^27^ the authors reported that increased intrauterine retention time (ie, time between feticide and delivery for terminations of pregnancies; during which maceration occurs) was a significant factor for nondiagnostic brain PMUS (but not cardiac PMUS). Increasing gestational age and intracardiac injection during feticide were the only factors found to be related to nondiagnostic cardiac PMUS for the terminations of pregnancy. All of these studies widely suggest that increased maceration score is the most significant contributing factor for a nondiagnostic brain, cardiac, and whole body PMUS study.^27^, ^29^, ^30^

An interesting observation in our study was that in the univariable analyses, we found that gestational age, postmortem weight, CRL and CHL, HC, and certain modes of fetal demise (IUD and miscarriages) had an effect on the likelihood of a diagnostic PMUS image. Mode of fetal demise had a negative impact on image quality, possibly given that IUFDs and miscarriages were more likely to be associated with a prolonged intrauterine retention rate and thus maceration severity score.

The fetal size factors had a negative effect on the diagnostic quality of brain PMUS but positive association for cardiac PMUS. For cardiac PMUS, we postulate that an increase in patient size (and thus cardiac size) may have allowed for better delineation of anatomical structures and thus a diagnostic result. One explanation could be that older fetuses are less likely to deform under gentle pressure from the ultrasound probe, causing less displacement of intrathoracic structures. This may explain the lower nondiagnostic PMUS rates for the heart and brain reported by Votino et al,^28^ who used formalin fixation prior to ultrasound examination.

It is harder to explain how the increase in gestational age and fetal size would adversely affect brain PMUS, but the smaller size of the cranial fontanelles, overlapping of the cranial sutures and scalp oedema following difficult extraction,^31^ may all contribute to the poorer sonographic penetrance required for adequate imaging (as previously shown in Figure 2). In addition, our use of a single linear high‐frequency ultrasound probe rather than one with a smaller footprint and lower frequency may have impacted our findings given the difficulty in adequately assessing extra‐axial spaces and reduced depth of sonographic penetration. Our finding is in contrast to a previous study assessing body weight limits for diagnostic quality postmortem MRI (PMMR),^32^ where it was reported that 90% of perinatal brain PMMR examinations were of diagnostic quality if a fetus weighed 301 g (or 415 g for whole body PMMR), compared with a brain PMMR diagnostic rate of 50% for those weighing 90 g (or 156 g for whole body PMMR). This is unsurprising given that ultrasound and MRI use different techniques to generate images, but it does suggest that PMMR would be an appropriate alternative method for larger fetuses where PMUS is nondiagnostic or limited by probe availability.

There is now also evidence that where a fetal brain malformation is suspected and termination of pregnancy is to be sought that intrauterine fetal MRI may be even more accurate than the PMMR.^33^ However, where an intrauterine death or stillbirth has occurred prior to the possibility for performing an intrauterine fetal MRI, then PMMR would be the next best option. There are currently no studies that have assessed the impact of diagnostic PMMR image quality in relation to different degrees of maceration (only that both autopsy and PMMR have a high agreement rate^21^) nor how PMMR diagnostic image quality changes with postmortem interval.

Another interesting finding in the present study was that PMI (time from delivery to PMUS) was not a significant factor for diagnostic image quality. This is highly relevant to clinical practice, particularly where specialist children's services and hospitals may not have their own maternity unit on‐site but could be referred fetal and neonatal deaths for specialist autopsy opinion. The transfer of the fetuses can sometimes incur delays because of logistical and transport issues, beyond the control of the referring clinicians. It is reassuring to find that this does not play a significant role in image quality, particularly where parents may wish for a NIA only, and therefore relevant when developing a post‐mortem imaging protocol where recommendations for optimal timing of examinations are important. As described above, the rapid tissue degradation that occurs during a prolonged intrauterine retention period (maceration) appears to play a more significant role in the diagnostic quality of the PMUS.

Our study had inherent limitations. We did not have autopsy correlation in all of our cases, since many cases did not consent to standard autopsy in addition to post‐mortem imaging, and therefore, we cannot compare nondiagnostic quality at PMUS against nondiagnostic rates at autopsy, although this was not the aim of the study.

Our study cohort was also skewed towards fetuses of a lower gestational age and lower post‐mortem weight, because of our recruitment/referral area. Because of lack of detailed information in our autopsy referral documentation regarding the mode of termination of pregnancy in some of our cases, it was also not possible to assess whether the use of intracradiac potassium chloride or lignocaine injection during feticide may have contributed to advanced maceration or poor diagnostic quality imaging, particularly for cardiac ultrasound in certain cases.

We were also limited with the ultrasound equipment available, where we used a single high‐frequency linear probe, which may have been less effective for deeper penetration of sonographic waves in larger fetuses where other probes (eg, microconvex array probe, with lower bandwith [4‐10 MHz] and a smaller footprint for the reduced size in fontanelles of larger fetuses and neonates) might have improved imaging. As such, our results for brain imaging are limited to the use of a high‐frequency linear probe in this setting, which could also account for the conflicting results in diagnostic imaging quality with increasing post‐mortem body weight. We also recognise the subjective nature of defining imaging as diagnostic or nondiagnostic, which generates reporter variability between centres, but this issue is inherent to all imaging studies reporting diagnostic yield.

In conclusion, whole body PMUS imaging is of diagnostic quality in over two‐thirds of fetal and neonatal deaths and would be a useful first line imaging tool in the majority of cases, apart from those with severe maceration. Cardiac and brain examinations are the most likely body parts to be nondiagnostic, with fetal size and gestational age also being significant contributory factors. A high throughput service could be developed around using PMUS in the first instance, prioritising PMMR for macerated fetuses (particularly with antenatal diagnosis of brain or cardiac abnormality) or where PMUS was subsequently found to be nondiagnostic.

## CONFLICT OF INTEREST

The authors declare no conflicts of interest.

## DATA ACCESSIBILITY

The data that support the findings of this study are available on request from the corresponding author. The data are not publicly available due to privacy or ethical restrictions.

## A. REPORTING TEMPLATE FOR POST‐MORTEM ULTRASOUND REPORT

Where applicable state normal, abnormal (with abnormality), or not examined for each body part reviewed.

**Table:** 

Patient Name:	
Patient Date of Birth:	
Patient Hospital ID:	
Date of Imaging Examination:	
Gestational Age:	
Mode of death/demise:	
Clinical Indication:	
**Neurological:**	
Cerebral hemispheres:	
Ventricular appearances:	
Sulcation pattern:	
Cavum septum pellucidum:	
Corpus callosum:	
Thalamus:	
Brainstem:	
Cerebellum/Vermis:	
Posterior fossa:	
Spinal cord:	
**Neck:**	
Thyroid:	
Thymus:	
**Thorax:**	
Airways (trachea and main bronchi):	
Right lung parenchyma/lobulation:	
Left lung parenchyma/lobulation:	
Right hemi‐diaphragm:	
Left hemi‐diaphragm:	
**Cardiac:**	
Cardiac axis:	
Cardiac size:	
Cardiac chambers:	
Septal Defects:	
Outflow tracts (LVOT/RVOT):	
Systemic veins:	
Pulmonary veins:	
**Abdomen:**	
Liver:	
Gallbladder:	
Pancreas:	
Spleen:	
Adrenals:	
Kidneys (right/left):	
Urinary Bladder:	
Stomach:	
Bowel rotation:	
Bowel appearance:	
Genitalia:	
Abdominal wall:	
**MSK/Soft Tissue:**	
Digits:	
Vertebrae:	
Soft tissue masses:	
**Conclusions (including suggestions for any further PM imaging):**	
